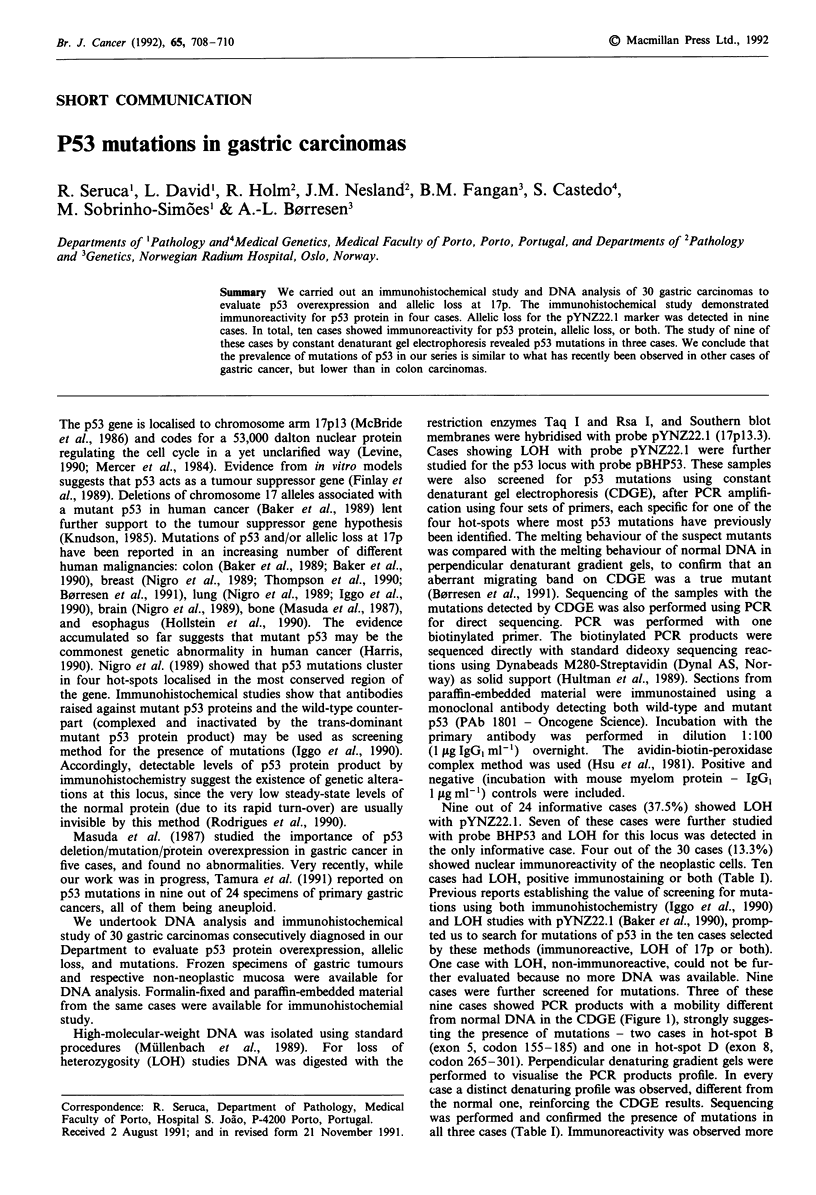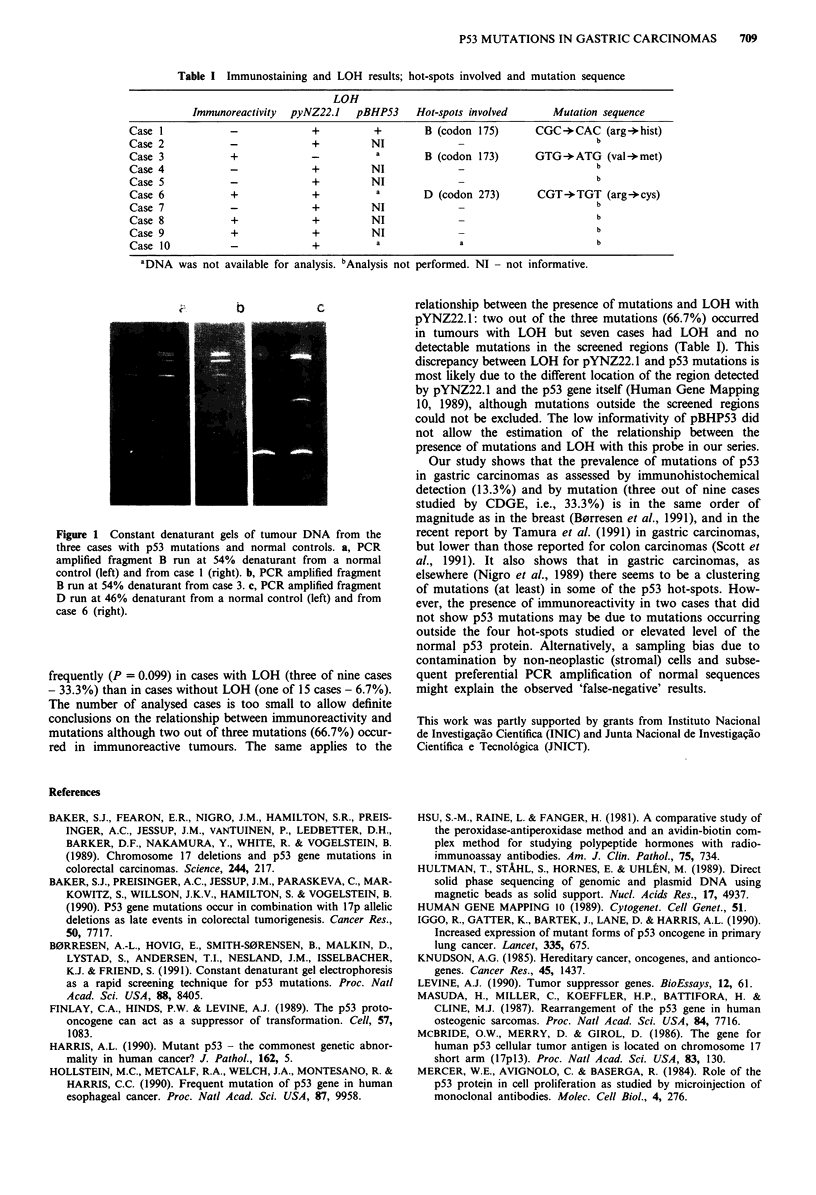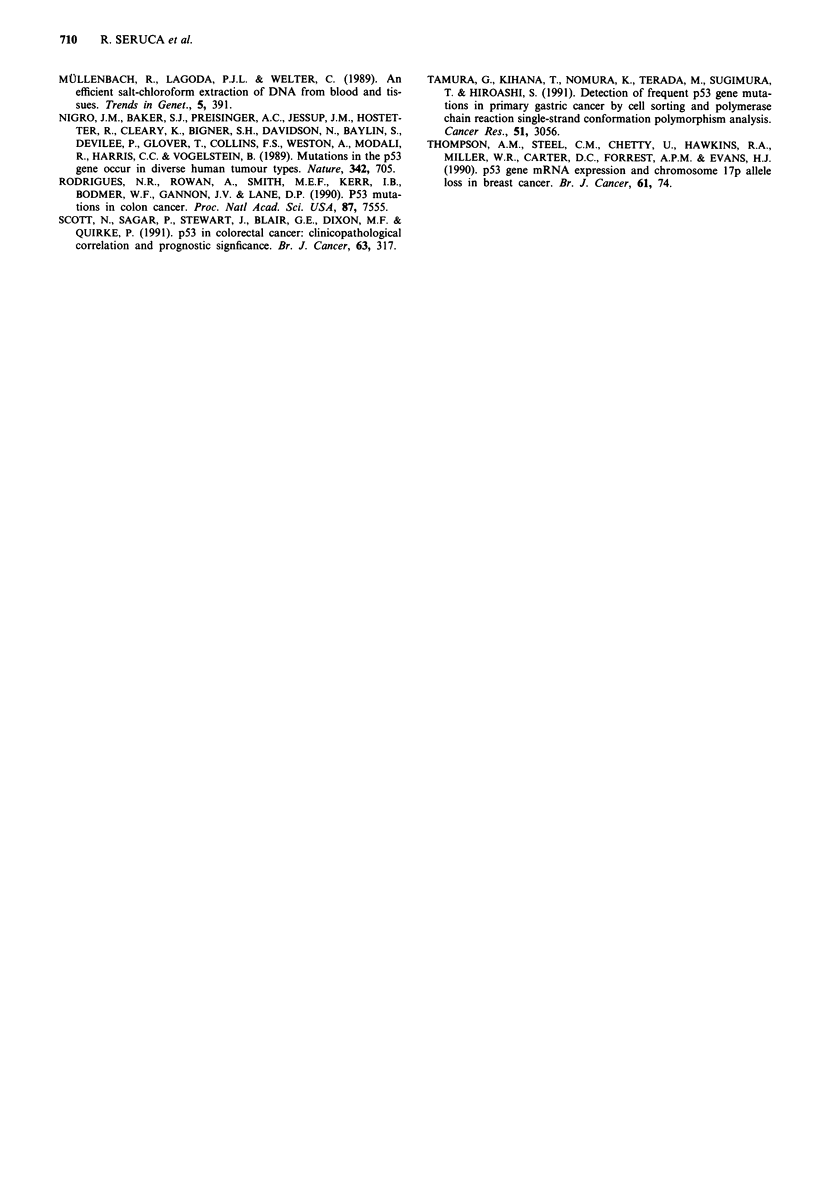# P53 mutations in gastric carcinomas.

**DOI:** 10.1038/bjc.1992.149

**Published:** 1992-05

**Authors:** R. Seruca, L. David, R. Holm, J. M. Nesland, B. M. Fangan, S. Castedo, M. Sobrinho-Simões, A. L. Børresen

**Affiliations:** Department of Pathology, Medical Faculty of Porto, Portugal.

## Abstract

**Images:**


*Br. J. Cancer* (1992), **65**, 708–710 © Macmillan Press Ltd., 1992

SHORT COMMUNICATION

### P53 mutations in gastric carcinomas

R. Seruca[1], L. David[1], R. Holm[2], J.M. Nesland[2], B.M. Fangan[3], S. Castedo[4], M. Sobrinho-Simões[1] & A.-L. Børresen[3]

*Departments of [1]Pathology and[4]Medical Genetics, Medical Faculty of Porto, Porto, Portugal, and Departments of [2]Pathology and [3]Genetics, Norwegian Radium Hospital, Oslo, Norway.*

**Summary** We carried out an immunohistochemical study and DNA analysis of 30 gastric carcinomas to evaluate p53 overexpression and allelic loss at 17p. The immunohistochemical study demonstrated immunoreactivity for p53 protein in four cases. Allelic loss for the pYNZ22.1 marker was detected in nine cases. In total, ten cases showed immunoreactivity for p53 protein, allelic loss, or both. The study of nine of these cases by constant denaturant gel electrophoresis revealed p53 mutations in three cases. We conclude that the prevalence of mutations of p53 in our series is similar to what has recently been observed in other cases of gastric cancer, but lower than in colon carcinomas.

The p53 gene is localised to chromosome arm 17p13 (McBride *et al.*, 1986) and codes for a 53,000 dalton nuclear protein regulating the cell cycle in a yet unclarified way (Levine, 1990; Mercer *et al.*, 1984). Evidence from *in vitro* models suggests that p53 acts as a tumour suppressor gene (Finlay *et al.*, 1989). Deletions of chromosome 17 alleles associated with a mutant p53 in human cancer (Baker *et al.*, 1989) lent further support to the tumour suppressor gene hypothesis (Knudson, 1985). Mutations of p53 and/or allelic loss at 17p have been reported in an increasing number of different human malignancies: colon (Baker *et al.*, 1989; Baker *et al.*, 1990), breast (Nigro *et al.*, 1989; Thompson *et al.*, 1990; Børresen *et al.*, 1991), lung (Nigro *et al.*, 1989; Iggo *et al.*, 1990), brain (Nigro *et al.*, 1989), bone (Masuda *et al.*, 1987), and esophagus (Hollstein *et al.*, 1990). The evidence accumulated so far suggests that mutant p53 may be the commonest genetic abnormality in human cancer (Harris, 1990). Nigro *et al.* (1989) showed that p53 mutations cluster in four hot-spots localised in the most conserved region of the gene. Immunohistochemical studies show that antibodies raised against mutant p53 proteins and the wild-type counterpart (complexed and inactivated by the trans-dominant mutant p53 protein product) may be used as screening method for the presence of mutations (Iggo *et al.*, 1990). Accordingly, detectable levels of p53 protein product by immunohistochemistry suggest the existence of genetic alterations at this locus, since the very low steady-state levels of the normal protein (due to its rapid turn-over) are usually invisible by this method (Rodrigues *et al.*, 1990).

Masuda *et al.* (1987) studied the importance of p53 deletion/mutation/protein overexpression in gastric cancer in five cases, and found no abnormalities. Very recently, while our work was in progress, Tamura *et al.* (1991) reported on p53 mutations in nine out of 24 specimens of primary gastric cancers, all of them being aneuploid.

We undertook DNA analysis and immunohistochemical study of 30 gastric carcinomas consecutively diagnosed in our Department to evaluate p53 protein overexpression, allelic loss, and mutations. Frozen specimens of gastric tumours and respective non-neoplastic mucosa were available for DNA analysis. Formalin-fixed and paraffin-embedded material from the same cases were available for immunohistochemial study.

High-molecular-weight DNA was isolated using standard procedures (Müllenbach *et al.*, 1989). For loss of heterozygosity (LOH) studies DNA was digested with the restriction enzymes Taq I and Rsa I, and Southern blot membranes were hybridised with probe pYNZ22.1 (17p13.3). Cases showing LOH with probe pYNZ22.1 were further studied for the p53 locus with probe pBHP53. These samples were also screened for p53 mutations using constant denaturant gel electrophoresis (CDGE), after PCR amplification using four sets of primers, each specific for one of the four hot-spots where most p53 mutations have previously been identified. The melting behaviour of the suspect mutants was compared with the melting behaviour of normal DNA in perpendicular denaturant gradient gels, to confirm that an aberrant migrating band on CDGE was a true mutant (Børresen *et al.*, 1991). Sequencing of the samples with the mutations detected by CDGE was also performed using PCR for direct sequencing. PCR was performed with one biotinylated primer. The biotinylated PCR products were sequenced directly with standard dideoxy sequencing reactions using Dynabeads M280-Streptavidin (Dynal AS, Norway) as solid support (Hultman *et al.*, 1989). Sections from paraffin-embedded material were immunostained using a monoclonal antibody detecting both wild-type and mutant p53 (PAb 1801 – Oncogene Science). Incubation with the primary antibody was performed in dilution 1:100 ($1\ \mu g\ IgG_1\ ml^{-1}$) overnight. The avidin-biotin-peroxidase complex method was used (Hsu *et al.*, 1981). Positive and negative (incubation with mouse myelom protein – $IgG_1$ $1\ \mu g\ ml^{-1}$) controls were included.

Nine out of 24 informative cases (37.5%) showed LOH with pYNZ22.1. Seven of these cases were further studied with probe BHP53 and LOH for this locus was detected in the only informative case. Four out of the 30 cases (13.3%) showed nuclear immunoreactivity of the neoplastic cells. Ten cases had LOH, positive immunostaining or both (Table I). Previous reports establishing the value of screening for mutations using both immunohistochemistry (Iggo *et al.*, 1990) and LOH studies with pYNZ22.1 (Baker *et al.*, 1990), prompted us to search for mutations of p53 in the ten cases selected by these methods (immunoreactive, LOH of 17p or both). One case with LOH, non-immunoreactive, could not be further evaluated because no more DNA was available. Nine cases were further screened for mutations. Three of these nine cases showed PCR products with a mobility different from normal DNA in the CDGE (Figure 1), strongly suggesting the presence of mutations – two cases in hot-spot B (exon 5, codon 155–185) and one in hot-spot D (exon 8, codon 265–301). Perpendicular denaturing gradient gels were performed to visualise the PCR products profile. In every case a distinct denaturing profile was observed, different from the normal one, reinforcing the CDGE results. Sequencing was performed and confirmed the presence of mutations in all three cases (Table I). Immunoreactivity was observed more

Correspondence: R. Seruca, Department of Pathology, Medical Faculty of Porto, Hospital S. João, P-4200 Porto, Portugal.
Received 2 August 1991; and in revised form 21 November 1991.

**Table I** Immunostaining and LOH results; hot-spots involved and mutation sequence

| | Immunoreactivity | LOH pyNZ22.1 | LOH pBHP53 | Hot-spots involved | Mutation sequence |
|---|---|---|---|---|---|
| Case 1 | – | + | + | B (codon 175) | CGC→CAC (arg→hist) |
| Case 2 | – | + | NI | – | [b] |
| Case 3 | + | – | [a] | B (codon 173) | GTG→ATG (val→met) |
| Case 4 | – | + | NI | – | [b] |
| Case 5 | – | + | NI | – | [b] |
| Case 6 | + | + | [a] | D (codon 273) | CGT→TGT (arg→cys) |
| Case 7 | – | + | NI | – | [b] |
| Case 8 | + | + | NI | – | [b] |
| Case 9 | + | + | NI | – | [b] |
| Case 10 | – | + | [a] | [a] | [b] |

[a]DNA was not available for analysis. [b]Analysis not performed. NI – not informative.

**Figure 1** Constant denaturant gels of tumour DNA from the three cases with p53 mutations and normal controls. **a**, PCR amplified fragment B run at 54% denaturant from a normal control (left) and from case 1 (right). **b**, PCR amplified fragment B run at 54% denaturant from case 3. **c**, PCR amplified fragment D run at 46% denaturant from a normal control (left) and from case 6 (right).

frequently ($P = 0.099$) in cases with LOH (three of nine cases – 33.3%) than in cases without LOH (one of 15 cases – 6.7%). The number of analysed cases is too small to allow definite conclusions on the relationship between immunoreactivity and mutations although two out of three mutations (66.7%) occurred in immunoreactive tumours. The same applies to the relationship between the presence of mutations and LOH with pYNZ22.1: two out of the three mutations (66.7%) occurred in tumours with LOH but seven cases had LOH and no detectable mutations in the screened regions (Table I). This discrepancy between LOH for pYNZ22.1 and p53 mutations is most likely due to the different location of the region detected by pYNZ22.1 and the p53 gene itself (Human Gene Mapping 10, 1989), although mutations outside the screened regions could not be excluded. The low informativity of pBHP53 did not allow the estimation of the relationship between the presence of mutations and LOH with this probe in our series.

Our study shows that the prevalence of mutations of p53 in gastric carcinomas as assessed by immunohistochemical detection (13.3%) and by mutation (three out of nine cases studied by CDGE, i.e., 33.3%) is in the same order of magnitude as in the breast (Børresen *et al.*, 1991), and in the recent report by Tamura *et al.* (1991) in gastric carcinomas, but lower than those reported for colon carcinomas (Scott *et al.*, 1991). It also shows that in gastric carcinomas, as elsewhere (Nigro *et al.*, 1989) there seems to be a clustering of mutations (at least) in some of the p53 hot-spots. However, the presence of immunoreactivity in two cases that did not show p53 mutations may be due to mutations occurring outside the four hot-spots studied or elevated level of the normal p53 protein. Alternatively, a sampling bias due to contamination by non-neoplastic (stromal) cells and subsequent preferential PCR amplification of normal sequences might explain the observed 'false-negative' results.

This work was partly supported by grants from Instituto Nacional de Investigação Científica (INIC) and Junta Nacional de Investigação Científica e Tecnológica (JNICT).

### References

BAKER, S.J., FEARON, E.R., NIGRO, J.M., HAMILTON, S.R., PREISINGER, A.C., JESSUP, J.M., VANTUINEN, P., LEDBETTER, D.H., BARKER, D.F., NAKAMURA, Y., WHITE, R. & VOGELSTEIN, B. (1989). Chromosome 17 deletions and p53 gene mutations in colorectal carcinomas. *Science*, **244**, 217.

BAKER, S.J., PREISINGER, A.C., JESSUP, J.M., PARASKEVA, C., MARKOWITZ, S., WILLSON, J.K.V., HAMILTON, S. & VOGELSTEIN, B. (1990). P53 gene mutations occur in combination with 17p allelic deletions as late events in colorectal tumorigenesis. *Cancer Res.*, **50**, 7717.

BØRRESEN, A.-L., HOVIG, E., SMITH-SØRENSEN, B., MALKIN, D., LYSTAD, S., ANDERSEN, T.I., NESLAND, J.M., ISSELBACHER, K.J. & FRIEND, S. (1991). Constant denaturant gel electrophoresis as a rapid screening technique for p53 mutations. *Proc. Natl Acad. Sci. USA*, **88**, 8405.

FINLAY, C.A., HINDS, P.W. & LEVINE, A.J. (1989). The p53 proto-oncogene can act as a suppressor of transformation. *Cell*, **57**, 1083.

HARRIS, A.L. (1990). Mutant p53 – the commonest genetic abnormality in human cancer? *J. Pathol.*, **162**, 5.

HOLLSTEIN, M.C., METCALF, R.A., WELCH, J.A., MONTESANO, R. & HARRIS, C.C. (1990). Frequent mutation of p53 gene in human esophageal cancer. *Proc. Natl Acad. Sci. USA*, **87**, 9958.

HSU, S.-M., RAINE, L. & FANGER, H. (1981). A comparative study of the peroxidase-antiperoxidase method and an avidin-biotin complex method for studying polypeptide hormones with radioimmunoassay antibodies. *Am. J. Clin. Pathol.*, **75**, 734.

HULTMAN, T., STÅHL, S., HORNES, E. & UHLÉN, M. (1989). Direct solid phase sequencing of genomic and plasmid DNA using magnetic beads as solid support. *Nucl. Acids Res.*, **17**, 4937.

HUMAN GENE MAPPING 10 (1989). *Cytogenet. Cell Genet.*, **51**.

IGGO, R., GATTER, K., BARTEK, J., LANE, D. & HARRIS, A.L. (1990). Increased expression of mutant forms of p53 oncogene in primary lung cancer. *Lancet*, **335**, 675.

KNUDSON, A.G. (1985). Hereditary cancer, oncogenes, and antioncogenes. *Cancer Res.*, **45**, 1437.

LEVINE, A.J. (1990). Tumor suppressor genes. *BioEssays*, **12**, 61.

MASUDA, H., MILLER, C., KOEFFLER, H.P., BATTIFORA, H. & CLINE, M.J. (1987). Rearrangement of the p53 gene in human osteogenic sarcomas. *Proc. Natl Acad. Sci. USA*, **84**, 7716.

MCBRIDE, O.W., MERRY, D. & GIROL, D. (1986). The gene for human p53 cellular tumor antigen is located on chromosome 17 short arm (17p13). *Proc. Natl Acad. Sci. USA*, **83**, 130.

MERCER, W.E., AVIGNOLO, C. & BASERGA, R. (1984). Role of the p53 protein in cell proliferation as studied by microinjection of monoclonal antibodies. *Molec. Cell Biol.*, **4**, 276.

MÜLLENBACH, R., LAGODA, P.J.L. & WELTER, C. (1989). An efficient salt-chloroform extraction of DNA from blood and tissues. *Trends in Genet.*, **5**, 391.

NIGRO, J.M., BAKER, S.J., PREISINGER, A.C., JESSUP, J.M., HOSTETTER, R., CLEARY, K., BIGNER, S.H., DAVIDSON, N., BAYLIN, S., DEVILEE, P., GLOVER, T., COLLINS, F.S., WESTON, A., MODALI, R., HARRIS, C.C. & VOGELSTEIN, B. (1989). Mutations in the p53 gene occur in diverse human tumour types. *Nature*, **342**, 705.

RODRIGUES, N.R., ROWAN, A., SMITH, M.E.F., KERR, I.B., BODMER, W.F., GANNON, J.V. & LANE, D.P. (1990). P53 mutations in colon cancer. *Proc. Natl Acad. Sci. USA*, **87**, 7555.

SCOTT, N., SAGAR, P., STEWART, J., BLAIR, G.E., DIXON, M.F. & QUIRKE, P. (1991). p53 in colorectal cancer: clinicopathological correlation and prognostic signficance. *Br. J. Cancer*, **63**, 317.

TAMURA, G., KIHANA, T., NOMURA, K., TERADA, M., SUGIMURA, T. & HIROASHI, S. (1991). Detection of frequent p53 gene mutations in primary gastric cancer by cell sorting and polymerase chain reaction single-strand conformation polymorphism analysis. *Cancer Res.*, **51**, 3056.

THOMPSON, A.M., STEEL, C.M., CHETTY, U., HAWKINS, R.A., MILLER, W.R., CARTER, D.C., FORREST, A.P.M. & EVANS, H.J. (1990). p53 gene mRNA expression and chromosome 17p allele loss in breast cancer. *Br. J. Cancer*, **61**, 74.